# K_2_P Channels as Key Regulators of Cardiovascular and Pulmonary Vascular Function

**DOI:** 10.3390/ph19040533

**Published:** 2026-03-25

**Authors:** Hala Y. Abdelnasser, Xinchun Pi, Lavannya M. Pandit, Bradley K. McConnell

**Affiliations:** 1 Department of Pharmacological and Pharmaceutical Sciences, College of Pharmacy, University of Houston, Houston, TX 77204-5037, USA; habdelna@cougarnet.uh.edu; 2 Department of Medicine, Baylor College of Medicine, Houston, TX 77030, USA; xinchun.pi@bcm.edu; 3 Pulmonary, Critical Care, and Sleep Medicine, Baylor College of Medicine, Michael E. DeBakey Veterans Affairs Medical Center, Houston, TX 77030, USA; lpandit@bcm.edu

**Keywords:** K_2_P, TASK, TWIK, pulmonary hypertension, GPCR

## Abstract

Two-pore domain potassium (K_2_P) channels are the most recently identified family of potassium channels. They are regarded as the largest group of background “leak” channels, encoded by 15 mammalian KCNK genes, and divided into six subfamilies (TWIK, TREK, TASK, TALK, THIK, and TRESK). These channels have a role in stabilizing the resting membrane potential. Their widespread presence in the heart and vasculature supports cellular homeostasis by regulating cardiac rhythm, vascular tone, and protection against ischemic stress. The TASK, TWIK, and TREK subfamilies are the most abundantly expressed K_2_P channel subfamilies in the cardiovascular system, and dysregulation of specific members has been strongly linked to the development of major cardiovascular diseases. Mutations in TASK-1 have been identified in patients with pulmonary arterial hypertension, providing human genetic evidence linking K_2_P dysfunction to pulmonary vascular disease. While alterations in other K_2_P channels, such as TREK-1, have been demonstrated in preclinical studies where reduced channel activity is associated with ischemia–reperfusion injury and promotes cardiac arrhythmias. Growing evidence suggests that K_2_P channels could serve as promising therapeutic targets, with pharmacological activation of TASK-1 and TREK-1, for instance, that might help restore vascular tone, reduce remodeling, and offer cardioprotection. Their unique leak-channel properties enable the development of highly selective treatments. This review addresses the molecular biology, physiological roles, and disease relevance of K_2_P channels in the cardiovascular and pulmonary systems, emphasizing their potential as targets for innovative therapies in cardiovascular diseases.

## 1. Introduction

K_2_P channels are the most recently discovered potassium channels and represent the most prominent family of background or “leak” potassium channels. They are encoded by 15 mammalian KCNK genes, grouped into six subfamilies based on sequence and functional similarity: TWIK, TREK, TASK, TALK, THIK, and TRESK [1]. K_2_P channels are expressed across both nervous and non-nervous tissues, with expression levels ranging from high—such as TWIK channels in the nervous system—to relatively low, as observed with TALK channels in the pancreas [2]. Unlike other potassium channels, K_2_P channels don’t have a voltage sensor, and they are not gated by the membrane potential; they generate constitutive background K currents [3]. Their tissue-specific expression and modulation by physiological stimuli such as pH, hypoxia, and temperature make them attractive therapeutic targets for cardiovascular diseases [4].

The name of the K_2_P family derives from their distinctive structure, which consists of four transmembrane segments and two pore domains (4TM/2P) [1]. The functional channels form dimers that create pseudo-tetrameric K^+^-selective pores. In terms of their electrophysiological properties, these channels generate time-independent background K^+^ currents that lack the voltage-dependent activation and inactivation kinetics characteristic of classical voltage-gated potassium channels. As a result, their likelihood of opening remains constant across different membrane potentials, designating them as “leak” K^+^ channels [3,5]. Although these channels act as constitutively active leak channels, they are regulated by various stimuli depending on their structure, including extracellular pH, G-protein-coupled receptors (GPCR), hypoxia, and mechanical stretch [4,6,7].

## 2. Molecular Biology: General Characteristics of K_2_P Channel Subfamilies

### 2.1. TWIK (Tandem of P Domains in Weak Inward Rectifier K^+^ Channel)

The TWIK subfamily has three members (encoded by three distinct genes): TWIK-1 is encoded by *KCNK1*, TWIK-2 by *KCNK6*, and K_2_P7.1 by *KCNK7* [1]. TWIK-1 was the first mammalian K_2_P channel member to be discovered [6]. TWIK-1 and TWIK-2 share 34% amino acid identity [7]. TWIK-1 mRNA is highly expressed in human tissues such as the heart and overlaps with TWIK-2 in most organs except the brain, where TWIK-2 is not expressed [3]. Both TWIK-2, and TWIK-1, display inwardly rectifying macroscopic current-voltage (IV) relationships under symmetrical potassium conditions, making them unique among mammalian K_2_P channels [1]. Additionally, TWIK-2 is distinguished from other K_2_P channels by its small single-channel conductance [7]. The TWIK subgroup activity is downregulated by intracellular acidosis [8]. Like other K_2_P channels, they have a phosphorylation site for protein kinase on their C-terminus, which regulates their function through cell signaling [6]. Meanwhile, the predominantly neuronal *KCNK7* channel is considered a silent K_2_P channel, as it has not shown measurable potassium conductance in heterologous expression systems, and exhibits very low expression in the cardiac system [1,9]. In the cardiovascular and pulmonary systems, TWIK channels contribute to the regulation of membrane potential and cellular excitability in vascular smooth muscle cells and endothelial cells (ECs) [10]. In particular, TWIK-2 has been implicated in maintaining vascular tone and endothelial function, and its deficiency has been associated with pulmonary vascular dysfunction and increased susceptibility to pulmonary hypertension in experimental models [11].

### 2.2. TREK (TWIK-Related K^+^ Channel)

The TREK subfamily includes TREK-1 that is encoded by *KCNK2*, TREK-2 by *KCNK10*, and TRAAK (TWIK-Related Arachidonic Acid-Stimulated K^+^ Channel) encoded by *KCNK4*. They are known as background K^+^ channels with a weakly voltage-dependent open probability that is influenced by both physical and chemical stimuli [7,12]. This subfamily is important not only for regulating membrane excitability and maintaining the resting membrane potential but also for contributing to metabolic regulation and sensory transduction processes, including thermo-sensitivity, nociception, chemosensitivity, and neuroprotection [12]. Adding on, both TREK-1 and TREK-2 have been detected in the heart, and the β-receptor antagonist carvedilol was able to block TREK-1 and TREK-2; this supports the role of the TREK family as an antiarrhythmic drug target [13,14,15].

### 2.3. TASK (TWIK-Related Acid-Sensitive K^+^ Channel)

The TASK subfamily includes TASK1, which is encoded by *KCNK3*, TASK3 by *KCNK9*, and TASK5 by *KCNK15*. Human TASK-1 mRNA is expressed in many organs, such as the pancreas, placenta, lung, and brain, with lower expression in the heart [7]. Additionally, TASK channels contribute to the oxygen-sensing machinery in lung neuroepithelial bodies, central neurons, and carotid bodies. They are also expressed in the central nervous system, implicating these channels in the use of volatile anesthesia [16]. TASK channels help regulate resting membrane potential and are sensitive to extracellular pH and oxygen levels [17]. TASK channels are K^+^-selective and exhibit outward rectification under low extracellular K^+^ conditions. Their rectification behavior follows the Goldman–Hodgkin–Katz (GHK) relationship and reflects the influence of K^+^ gradients rather than intrinsic voltage sensitivity [5].

### 2.4. TALK (TWIK-Related Alkaline pH-Activated K^+^ Channel Subfamily)

The alkaline-activated K_2_P subfamily consists of TASK-2 encoded by *KCNK5*, TALK-1 by *KCNK16*, and TALK-2 by *KCNK17*, all of which are highly sensitive to extracellular pH, displaying inhibition under acidic conditions and activation in alkaline environments [18]. The mRNA levels are highly expressed in epithelial organs such as the kidney and pancreas, where TALK channel expression is limited in the cardiovascular system [19].

### 2.5. THIK (Tandem-Pore Domain Halothane Inhibited the K^+^ Channel Subfamily)

This family includes THIK-1 which is encoded by *KCNK13* and THIK-2 by *KCNK12*. Although THIK-1 expression has not been documented in human tissues, THIK-2 is broadly expressed in the pancreas, heart, and skeletal muscle and exhibits inward rectification under physiological K^+^ distribution [18].

### 2.6. TRESK (TWIK-Related Spinal Cord K^+^ Channel Subfamily)

TRESK, encoded by *KCNK18*, is the most recently discovered K_2_P subfamily identified in the human genome and is expressed in the spinal cord and brain [14]. It is stimulated in response to a calcium signal through calcium/calmodulin-dependent protein phosphatase [15]. Unlike the TASK subfamily, TRESK is less sensitive to extracellular pH [20]. Their macroscopic current became more linear under symmetrical K^+^ conditions, while maintaining an outwardly rectifying profile [7]. Both THIK and TRESK channels have limited evidence that links them to cardiovascular or pulmonary physiology.

Those K_2_P channels function in the pulmonary vasculature to control the resting membrane potential of pulmonary arterial smooth muscle cells (PASMCs) and, to a lesser extent, of ECs [21]. They generate a constitutive outward K^+^ leak current to maintain a more negative (hyperpolarized) membrane potential and stabilize the resting membrane potential [1]. This background K^+^ conductance counterbalances the depolarizing inward current and limits non-selective cation entry or receptor-operated currents, thereby preventing the membrane potential from reaching the activation threshold of voltage-gated Ca^2+^ channels [22,23]. As a result, L-type and T-type Ca^2+^ channels remain predominantly closed under basal conditions, limiting Ca^2+^ influx and maintaining low intracellular Ca^2+^ concentrations. This electrical stabilization is essential for preserving pulmonary vascular smooth muscle quiescence, as even small depolarizations can markedly increase Ca^2+^ entry and activate Ca^2+^-dependent contractile and proliferative signaling pathways [24]. A similar membrane-stabilizing role of K_2_P channels is also observed in cardiac tissues, where these channels contribute to maintaining resting membrane potential and regulating cardiomyocyte excitability [2]. K_2_P channels, including TASK-1 and TREK-1, contribute to the repolarization phase of the cardiac action potential and regulate action potential duration rather than initiating the depolarizing phase of the action potential. Inhibition or genetic deletion of these channels leads to prolongation of action potential duration, a condition that can promote arrhythmogenesis. By mediating a constitutive outward K^+^ leak current, K_2_P channels help stabilize the resting membrane potential, which keeps it below the threshold required for initiating new action potentials. Adding on, K_2_P channels participate in electrical remodeling during cardiac diseases such as atrial fibrillation, where their dysfunction can alter action potential duration and increase the risk of proarrhythmic events [25,26,27].

## 3. Physiological Functions of K_2_P Channels in the Cardiovascular Systems and Their Involvement in Cardiac Diseases

**Figure 1:**
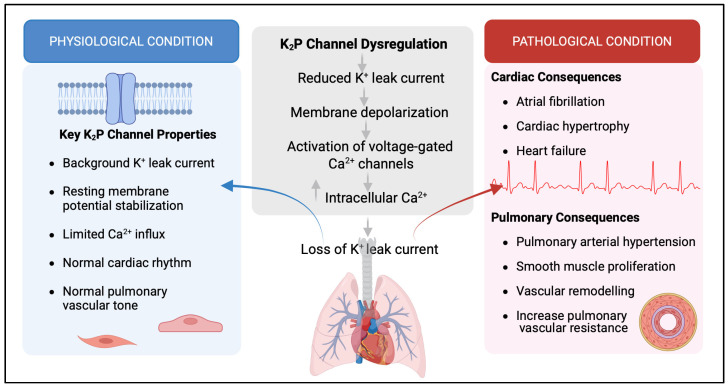
Role of K_2_P channels in cardiovascular and pulmonary vascular physiology and disease. Schematic illustrating the physiological and pathological roles of two-pore domain potassium (K_2_P) channels. Under physiological conditions (blue), K_2_P channels contribute to background K^+^ conductance, thereby regulating cardiac rhythm and atrial action potential duration, stabilizing the resting membrane potential, and limiting Ca^2+^ entry in PASMCs and ECs. In contrast, under pathological conditions (red), dysregulation of these channels disrupts membrane homeostasis, leading to electrical instability and cardiovascular disease, including atrial fibrillation, cardiac hypertrophy, and heart failure. In the pulmonary circulation, K_2_P channel dysfunction promotes membrane depolarization, increased Ca^2+^ signaling, and enhanced smooth muscle cell proliferation and migration, contributing to pulmonary arterial hypertension and vascular remodeling Created by Biorender.

K_2_P channels have a broad distribution in the cardiovascular system, underscoring their roles in regulating heart rhythm, mechanical stress, and cardioprotection [28]. The most common K_2_P channels in mammalian heart cells are TASK, TWIK, and TREK channels [2]. TWIK-1 is highly expressed in the atrium and Purkinje fibers [2]. Since TWIK-1 is widely spread in cardiac tissues, it helps control heart function by contributing to the cardiac inward rectifier K^+^ current. This current helps maintain the resting membrane potential and plays a key role in repolarization during the final step of the action potential [24]. TWIK-2, another member of the TWIK family, is prominent in the right atrium and acts as a voltage-gated K^+^ channel at depolarized potentials, affecting both early and late repolarization of the cardiac action potential [25]. A study measuring K_2_P channel levels during sinus rhythm showed that TWIK-1 and TASK-1 are present in both atria but are found at very low levels in the ventricles [29]. This indicates that both TWIK-1 and TASK-1 play important roles in atrial electrical activity and arrhythmias (Figure 1).

Additionally, K_2_P channel dysfunction has been implicated in various cardiac diseases such as ischemic injury, arrhythmias, and heart failure [9,30] (Table 1). Specifically, dysregulation of the TASK and TREK subfamilies contributes to arrhythmogenesis, atrial remodeling, atrial fibrillation (AF), and heart failure. AF is the most common sustained arrhythmia and is associated with high morbidity and mortality, characterized by disorganized, rapid electrical signals in the atria that produce chaotic heartbeats [31]. Importantly, TASK-1 current is one of the main factors in background conductance in human atrial cardiomyocytes, and its significance in the atria has been supported by various studies [32]. For instance, chronic AF is associated with long-term electrical remodeling of atrial cardiomyocytes and is linked to the elevation of TASK-1 expression by approximately 60% in the right atrium compared to individuals with sinus rhythm, leading to a threefold increase in current and shortening of the action potential. Pharmacological inhibition with the TASK-1-selective blocker A293 prevented this effect, confirming that TASK-1 contributes to the electrical changes observed in atrial fibrillation [29,33]. Therefore, TASK-1 channel inhibition is a promising treatment approach for AF. In contrast, perioperative AF, which occurs following thoracic surgery, is largely driven by acute inflammation and oxidative stress rather than long-term electrical remodeling, and studies have reported reduced TASK-1 expression in this context. Similarly, AF associated with heart failure develops in the setting of structural remodeling, fibrosis, and neurohormonal activation, factors that can alter ion channel expression and have been associated with decreased TASK-1 activity [34,35]. Experimental data using the monocrotaline-induced pulmonary hypertension rat model to explore TASK-1 involvement in atrial arrhythmia showed that selective TASK-1 inhibition (ML365) increased AF susceptibility, suggesting that loss of TASK-1 function may promote atrial arrhythmogenesis under certain pathological conditions [36].

Meanwhile, TASK-3 shares many similarities with TASK-1 and forms heterodimerized TASK-1/TASK-3 channels on the surface of atrial cardiomyocytes; it is unclear whether TASK-3 levels are affected in atrial fibrillation, but, since it can heterodimerize with TASK-1, it should be considered a pharmacological target [39]. In human cardiac tissue, TASK-1 expression is diminished in settings of myocardial stress, including cardiac hypertrophy and structural remodeling, irrespective of the presence of heart failure [30]. Experimental evidence further supports a functional role for this channel in cardiac pathology. In a cardiomyopathy mouse model, global TASK-1 deletion confers protection against pressure overload by attenuating hypertrophic remodeling and preserving cardiac function. In contrast, global deletion of TASK-3 produces an opposing phenotype, characterized by cardiac hypertrophy and a delayed onset of cardiac dysfunction. The cardioprotective effects associated with TASK-1 loss-of-function are thought to arise from enhanced physiological hypertrophic signaling and the preservation of metabolic homeostasis. Collectively, these findings indicate that pharmacological inhibition of TASK-1 may represent a promising therapeutic approach for mitigating cardiac dysfunction [48]. However, the TASK-1 inhibition consequences appear to be disease dependent. While TASK-1 suppression may provide protection against cardiac remodeling, reduced TASK-1 has also been accompanied by increased susceptibility to atrial arrhythmia in certain pathological conditions. Together, these findings underscore the complex role of TASK-1 in cardiac physiology, where it contributes to both electrophysiological regulation and structural remodeling, indicating that therapeutic targeting of this channel may have disease-specific benefits or risks.

Beyond AF and cardiac remodeling, whole-genome sequencing identified a gain-of-function mutation in TASK-4 in idiopathic ventricular fibrillation, resulting in a threefold increase in current that improved conduction by enhancing repolarization of the action potential, thereby promoting reentrant arrhythmias [40]. Another study found that atrial TWIK-1 expression is reduced in chronic atrial fibrillation, suggesting a role for TWIK-1 in electrical remodeling [41]. On the contrary, another study showed that TWIK-1 is upregulated in AF that is associated with valvular heart disease [49]. These seemingly opposing findings likely reflect differences in the underlying disease mechanisms, as chronic AF represents advanced electrical remodeling, whereas valvular AF develops in the context of pressure or volume overload and structural remodeling. In addition to the involvement of other K_2_P channels in cardiac disease, TREK-1 is mainly expressed in the ventricular myocardium of both humans and mice compared to the atrium. In patients with significantly reduced ventricular function, TREK-1 mRNA and protein levels are decreased, contributing to action potential prolongation [47]. In patients with chronic AF and concurrent heart failure, atrial TREK-1 mRNA expression decreases by more than 80%, with a greater reduction in the right atrium than in sinus rhythm controls, and this downregulation is associated with prolonged atrial effective refractory periods [44]. Moreover, TREK-1 dysregulation has been linked to the development of cardiac fibrosis, hypertrophy, and the progression of heart failure [9]. A study demonstrated that TREK-1 plays a key role in cardiac ischemia–reperfusion injury and remodeling after myocardial infarction, wherein in the TREK-1 knockout mouse model, infarct size increased after coronary ligation, suggesting that stimulating TREK-1 could offer cardioprotection against ischemia–reperfusion injury [43]. Another study showed that mice with global TREK-1 deletion exhibit exaggerated pressure overload-induced cardiac hypertrophy, with molecular signatures characteristic of a pathological cardiac phenotype [50]. Conversely, atrial TREK-2 (KCNK10) expression increases in heart failure, which may facilitate electrical reentry and promote atrial fibrillation [47]. This highlights the crucial role of TREK-1 in maintaining cardiac electrophysiology and protecting the myocardium under stress conditions. Pharmacological modulation of TREK-1 could have a promising therapeutic effect for cardiac remodeling and ischemia–reperfusion injury in patients with ischemic heart disease.

Collectively, these findings underline the crucial role of K_2_P channels in the development of atrial fibrillation and other chronic heart diseases. Since K_2_P channels are largely unaffected by traditional K^+^ channel blockers [4], the development of atrial-specific modulators is a promising treatment strategy that can control AF while reducing the risk of ventricular arrhythmia [51]. These findings underscore the importance of targeting K_2_P channels as a new therapeutic option for heart conditions.

## 4. Physiological Roles of K_2_P Channels in Pulmonary Vasculature and Their Contribution to Pulmonary Diseases

K_2_P channels serve as major determinants of pulmonary vascular excitability by providing a background potassium conductance that stabilizes the resting membrane potential and constrains voltage-dependent calcium entry in both PASMCs and ECs) (Figure 1) [1]. K_2_P channels regulate the vascular tone by sensing the pH. Specifically, they mediate smooth muscle depolarization during acidification and promote hyperpolarization during alkalization, linking pH changes to vascular tone control [52]. They also contribute to pulmonary vascular responses to hypoxia, where TASK-1 participates in oxygen-sensing pathways; in small pulmonary arteries, acute hypoxia inhibits TASK-1, causing depolarization and increasing Ca^2+^ influx [53]. All these factors underscore the importance of K_2_P channels in regulating the pulmonary vasculature; dysfunction or dysregulation of these channels can lead to the development of various diseases [54].

### 4.1. TREK-1 Role in Pulmonary Diseases

Hyperoxia, a condition where the lungs are exposed to abnormally high oxygen levels, is linked to lung injury and causes pathological remodeling of alveolar epithelial cells [46]. Experimental studies have shown that TREK-1 undergoes changes during hyperoxia, and its expression was decreased in mouse alveolar epithelial cells exposed to hyperoxia. This suggests that TREK-1 plays a role in regulating epithelial cell proliferation under harmful oxygen conditions [46].

Clinically, hospitalized patients requiring supplemental oxygen therapy for hypoxic respiratory failure are at risk of developing hyperoxia [51]. This highlights the need to understand the molecular mechanisms linking hyperoxia to epithelial dysfunction, with TREK-1 emerging as a significant potential mediator.

In vitro experiments showed that TREK-1 deficiency increased the production and secretion of monocyte chemoattractant protein-1 (MCP-1), a key chemokine involved in pulmonary inflammation. This effect was associated with increased phosphorylation of JNK1/2/3 isoforms, linking TREK-1 activity to hyperoxia-induced release of inflammatory mediators [55]. From a therapeutic standpoint, pharmacological activation of TREK-1 has demonstrated promising results in preclinical models. In a hyperoxia-induced mouse model, intratracheal administration of TREK-1 activators, including ML335 and BL1249, significantly reduced lung injury [56]. Treated animals showed improved lung architecture, reduced physiological impairment, and lower levels of biochemical markers of epithelial damage. Additionally, cytokine levels were decreased, indicating a broad protective effect of TREK-1 activation against hyperoxia-induced pulmonary injury [56].

Collectively, these studies emphasize the vital role of TREK-1 as a regulator of alveolar epithelial cell responses to hyperoxia, affecting both proliferation and inflammatory signaling. The therapeutic benefits seen with TREK-1 activators strongly support further investigation of the TREK-1 channel as a pharmacological target in managing hyperoxia-induced injury.

Meanwhile, influenza A virus is a major cause of pneumonia and acute respiratory distress syndrome (ARDS), which are leading causes of morbidity and mortality in developed countries [57]. Because inflammatory mediators are linked to changes in cell membrane potential, potassium channels such as TREK-1 have been investigated as modulators of influenza-induced lung injury [58]. In preclinical studies, infected mice treated with the TREK-1 activators BL1249 and ML335 showed significant improvements in multiple indicators of lung injury, including reductions in bronchoalveolar fluid total protein and decreased inflammatory cytokine levels [58]. Besides its protective role in alveolar epithelial cells, TREK-1 is also involved in innate immune signaling [59]. A recent study using primary alveolar macrophages from TREK-1 knockout mice demonstrated that TREK-1 regulates NLRP3 inflammasome activation and macrophage-mediated inflammatory responses during viral infection [59]. Collectively, these findings indicate that TREK-1 may play a protective role in limiting inflammation and lung injury during influenza infection.

Pulmonary hypertension is characterized as an increase in the mean pulmonary arterial pressure (mPAP) > 20 mmHg and an elevation in the pulmonary vascular resistance by >3 wood units [60]. It is classified into five distinct groups according to guidelines established by the World Symposium on Pulmonary Hypertension, based on clinical presentation, hemodynamic features, pathophysiological mechanisms, and therapeutic management [61]. Pulmonary arterial hypertension is classified as the first group, where there is an increase in the vascular resistance in the small pulmonary arteries that eventually might cause right heart failure [62,63]. TREK-1 (*KCNK2*), which is highly expressed in PASMCs, helps maintain the resting membrane potential and limits calcium entry, and is gaining importance in pulmonary arterial hypertension [45]. Pharmacological activation of TREK-1 has been shown to hyperpolarize PASMCs and inhibit calcium influx, thereby reducing vasoconstriction [45]. In pulmonary arterial hypertension, TREK-1 expression is decreased, although its channel function remains intact. Importantly, activating TREK-1 in pulmonary arterial hypertension mouse models restored the resting membrane potential and corrected abnormal calcium signaling [45]. Another study found that TREK-1 upregulation is associated with increased proliferation and migration of PASMCs, suggesting a role in vascular remodeling [64]. These findings indicate that TREK-1 may have both protective and harmful effects depending on the disease stage and vascular compartment.

### 4.2. TASK-1 (KCNK3) in Pulmonary Arterial Hypertension

Among the K_2_P channel family, TASK-1 (*KCNK3*) has emerged as the most strongly implicated in the development of pulmonary arterial hypertension. Heterozygous loss-of-function mutations in *KCNK3* were first identified as a genetic cause of heritable and idiopathic pulmonary arterial hypertension [37]. Whole-exome sequencing revealed six different heterozygous missense variants in *KCNK3* among 92 familial pulmonary arterial hypertension cases and 230 patients with idiopathic pulmonary arterial hypertension, and these mutations impair channel activity through various mechanisms, depending on their location; some are in the N-terminal domain, while others are in the pore domain, which is responsible for pH sensitivity [37]. The mechanisms include altered trafficking, defective channel assembly, impaired pH sensing, and disruption of potassium selectivity [37]. Additionally, two novel missense mutations in exon 2 of *KCNK3* have been reported in patients with both idiopathic and heritable pulmonary arterial hypertension, with homozygous carriers showing particularly aggressive disease phenotypes [65].

Meanwhile, two new mutations in TASK-1 have been identified in pulmonary arterial hypertension: G106R and L214R, which significantly decrease channel currents compared with wild-type TASK-1 when transiently expressed in a modified embryonic cell line [66]. The pharmacological activation at alkaline pH fails to restore channel activity in these mutants, confirming a fundamental loss of TASK-1 function [66]. Additionally, potassium channel mutations in pulmonary arterial hypertension represent a class of channelopathies in which the mutation location determines the mechanism of dysfunction, as the V221L TASK-1 mutation produces a pH-dependent loss of function [67].

Furthermore, animal models reinforce the role of TASK-1 dysfunction in pulmonary vascular remodeling, in which monocrotaline-induced PH rats showed diminished KCNK3 expression in both PASMCs and ECs, correlating with membrane depolarization [68]. Additionally, long-term inhibition of *KCNK3* promotes distal neomuscularization, increasing PASMCs, endothelial, and fibroblast proliferation, while pharmacological activation of TASK-1 helps restore hyperpolarization and alleviates pulmonary hypertension in vivo [68]. Moreover, another study shows that co-assembly of KCNK3 with KCNK9 (TASK-3) may provide partial protection against KCNK3 loss-of-function in vitro, suggesting a potential compensatory mechanism [67].

Together, these findings identify TASK-1 (KCNK3) as a key molecular factor in pulmonary arterial hypertension and a promising therapeutic target, and TASK-1 mutations as a cause of both heritable and idiopathic PAH.

### 4.3. TWIK-2 (KCNK6) in Pulmonary Hypertension

TWIK-2 is highly expressed in the vascular system and is among the most abundant members of the K_2_P subfamily [26]. TWIK-2 (*KCNK6*) has mainly been studied using genetic knockout models, in which TWIK-2 deficiency in mice causes pulmonary hypertension associated with vascular wall remodeling mediated by the Rho-kinase pathway, which inhibits myosin light chain phosphatase and helps maintain vascular smooth muscle contractility [11]. In this model, the pulmonary hypertension phenotype was eliminated by Fasudil, a Rho-kinase inhibitor, confirming that TWIK-2 influences vascular tone via Rho-kinase signaling [11]. Further research showed that TWIK-2 knockout mice exhibit exaggerated vasoconstriction in distal pulmonary vessels compared to proximal vessels. This response was associated with increased depolarization and hypercontractility upon stimulation with vasoconstrictors, such as phenylephrine and thromboxane A2 agonists [64]. These findings highlight that TWIK-2 differentially regulates contractile responses along the pulmonary vascular tree and contributes to microvascular hyperreactivity in pulmonary hypertension. Although these murine models provide important mechanistic insights into TWIK-2-mediated regulation of pulmonary vascular tone, the direct translation of these findings to human pulmonary arterial hypertension should be interpreted with caution due to species differences in pulmonary vascular physiology.

Taken together, these studies highlight the diverse roles of K_2_P channels in the development of pulmonary hypertension. TASK-1 (*KCNK3*) is the most affected K_2_P channel, supported by strong genetic and functional evidence linking its dysfunction to heritable and idiopathic pulmonary arterial hypertension. In contrast, TREK-1 (*KCNK2*) may contribute to pathogenesis or to an adaptive response, depending on disease conditions and the activated vascular compartment. The role of TREK-1 in mediating the pathogenesis of pulmonary arterial hypertension requires further investigation. Additionally, TWIK-2 (*KCNK6*) modulates pulmonary vascular contractility through Rho-kinase-dependent signaling and shows regional differences in response to vasoconstrictors, affecting mechanisms of membrane depolarization, calcium entry, and smooth muscle hyperproliferation. K_2_P channel dysfunction offers a unifying explanation for pulmonary vascular remodeling, and the pharmacological strategies that restore or enhance K_2_P channel activity could therefore represent a new class of targeted therapies for pulmonary hypertension.

## 5. Role of G-Protein-Coupled Receptor (GPCR) Signaling in Modulating K_2_P Channels During Pulmonary Hypertension

Pulmonary hypertension is characterized by vascular remodeling, persistent vasoconstriction, and increased pulmonary vascular resistance [69]. Endothelin-1 (ET-1) is a central vasoactive mediator in pulmonary hypertension. Clinical studies have consistently shown elevated ET-1 levels in the lungs of patients with pulmonary hypertension and pulmonary arterial hypertension mouse models, with levels correlating with disease severity. Therefore, Endothelin-A receptor (ET-A) antagonists are commonly used as first-line therapies for pulmonary hypertension. ET-1 exerts its effects mainly through the ET-A receptor, a GPCR that is highly expressed on PASMCs [37,65].

Upon binding to the ET-A receptor, ET-1 activates Gq/11-coupled signaling pathways, leading to stimulation of phospholipase C (PLC). PLC hydrolyzes phosphatidylinositol-4,5-bisphosphate to generate the second messenger diacylglycerol (DAG) and inositol-1,4,5-trisphosphate (IP_3_). IP_3_ induces the release of Ca^2+^ from the sarcoplasmic reticulum, while DAG activates protein kinase C (PKC), together resulting in elevated intracellular Ca^2+^ and downstream kinase signaling [37,70,71]. These events are critical for pulmonary artery smooth muscle cell contraction, proliferation, and migration, which drives pulmonary vascular remodeling, a central pathological feature in the progression of pulmonary hypertension [72].

**Figure 2:**
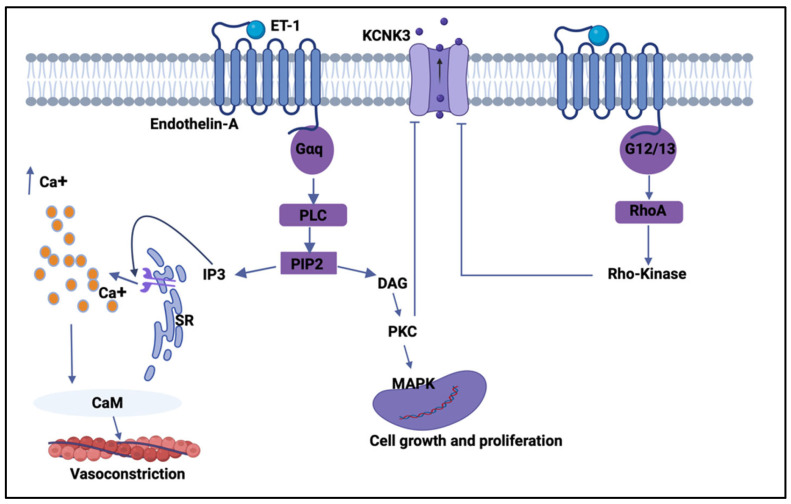
Endothelin-1-mediated GPCR signaling modulates TASK-1 (*KCNK3*) activity to regulate pulmonary vascular tone and remodeling. Schematic illustrating the signaling pathways activated by endothelin-1 (ET-1) binding to the endothelin-A (ET-A) receptor on pulmonary arterial smooth muscle cells. ET-A receptor activation engages Gαq, leading to phospholipase C (PLC)-dependent hydrolysis of phosphatidylinositol-4,5-bisphosphate (PIP_2_) into inositol-1,4,5-trisphosphate (IP_3_) and diacylglycerol (DAG). IP_3_ induces Ca^2+^ release from the sarcoplasmic reticulum (SR), elevating cytosolic Ca^2+^ levels and activating calmodulin (CaM)-dependent contractile machinery that promotes vasoconstriction. In parallel, DAG activates protein kinase C (PKC), which stimulates downstream MAPK signaling to drive gene transcription, cell growth, and proliferation. ET-A receptor signaling also couples to G12/13, activating RhoA and Rho-kinase pathways that further contribute to cytoskeletal remodeling and proliferative responses. Concomitantly, PKC- and Rho-kinase-dependent mechanisms inhibit TASK-1 (*KCNK3*) channel activity, reducing outward K^+^ currents, promoting membrane depolarization, and amplifying Ca^2+^ signaling. Created by Biorender.

Recent research has explored the interaction between ET-1 signaling and K_2_P potassium channels, especially the TASK-1 (*KCNK3*) subtype. A study confirmed that ET-1 depolarizes human PASMCs by phosphorylating the C-terminal domain of TASK-1 via PKC at clinically relevant concentrations, thereby reducing TASK-1 activity, causing membrane depolarization, calcium influx, and sustained contraction [73]. Another study found that ET-1 inhibits TASK-1 channel activity in smooth muscle cells through the Rho-kinase pathway (Figure 2) [68]. These findings suggest that ET-1-induced suppression of TASK-1 is a new pathological mechanism contributing to pulmonary hypertension and help explain why TASK-1 dysfunction is closely linked to the disease phenotype. Importantly, the regulation of K_2_P channels by GPCR pathways is not limited to pulmonary vascular remodeling. Studies have demonstrated that a single G-protein-coupled signaling pathway can exert divergent effects on members of the K_2_P potassium channel family in the neuronal system. Specifically, Gαq activation suppresses the activity of TASK and TREK channels while concurrently enhancing TRESK channel function [74]. As a result, the overall impact of Gαq signaling on background potassium conductance, and consequently on neuronal excitability, is difficult to anticipate since this outcome is determined by the relative abundance of individual K_2_P channel subtypes within a given neuron, as well as the extent to which downstream elements of the Gαq-dependent signaling cascade are engaged [74]. Collectively, these findings underscore the significance of K_2_P-ETA receptor interactions, and future therapies might target not only the ET-A receptor directly but also the downstream modulation of K_2_P channels to restore vascular balance.

## 6. Role of K_2_P Channels in Systemic Hypertension and Vascular Pathology

Systemic blood pressure (BP) is a complex trait influenced by genetic predisposition and environmental factors. At the vascular level, systolic BP regulation relies on the ability of small arterioles to modulate peripheral vascular resistance, and this process is mediated by cytosolic calcium for contraction and potassium channels for hyperpolarization [10]. Increasing evidence indicates that K_2_P channels play a crucial role in this regulation, linking vascular tone to systemic hypertension.

Meanwhile, TWIK-2 is highly expressed in blood vessels. TWIK-2 knockout mouse models have provided direct evidence of its role in systemic hypertension, as its deficiency promotes vascular dysfunction and increases blood pressure by causing smooth muscle depolarization through activation of the Rho kinase pathway [42]. In anesthetized TWIK-2 knockout mice, peripheral vascular resistance was significantly higher than in wild-type animals, while at the cellular level, freshly isolated aortic smooth muscle cells from TWIK-2 knockouts were more depolarized compared to controls [10].

Beyond TWIK-2, TASK-1 also contributes to systemic blood pressure regulation. Global TASK-1 knockout mice exhibit increased blood pressure, highlighting TASK-1’s role in maintaining vascular stability, and human data support this association, showing that TASK-1 genetic variants are associated with systemic hypertension. A single nucleotide polymorphism (SNP) in *KCNK3*, the gene encoding TASK-1, has been linked to mean arterial pressure. Furthermore, other *KCNK3* variants are related to plasma renin activity and aldosterone levels, linking TASK-1 dysfunction to changes in neurohormonal regulation of blood pressure [38,75].

## 7. Conclusions

K_2_P potassium channels have emerged as central regulators of membrane excitability, vascular tone, and tissue homeostasis in both the cardiovascular and pulmonary systems. By providing a constitutive background potassium conductance, K_2_P channels stabilize the resting membrane potential, restrain voltage-dependent calcium entry, and integrate mechanical, metabolic, and receptor-mediated signals. Their distinctive regulation by extracellular pH, hypoxia, and GPCR signaling distinguishes them from traditional K^+^ channels. This review highlights how dysregulation of key K_2_P subtypes, particularly TASK-1 (*KCNK3*), TREK-1 (*KCNK2*), and TWIK-2 (*KCNK6*), contributes to the pathogenesis of cardiac arrhythmias, pulmonary arterial hypertension, lung injury, and systemic vascular disease.

Importantly, K_2_P channels act as downstream effectors of GPCR signaling, particularly ET-1/ET-A receptor pathways in pulmonary hypertension. ET-1-mediated suppression of TASK-1 via PKC and Rho-kinase signaling provides a mechanistic link between vasoactive mediators, membrane depolarization, and vascular remodeling, offering new opportunities for therapeutic intervention beyond receptor antagonism.

## 8. Clinical Implications

The strong genetic, mechanistic, and pharmacological evidence reviewed here positions K_2_P channels as promising therapeutic targets in cardiopulmonary and vascular disease. TASK-1 inhibition may offer atrial-selective antiarrhythmic strategies. For instance, amiodarone may exert part of its anti-atrial fibrillation efficacy through inhibition of the atria-enriched two-pore domain TASK-1 background potassium channel [76]. Several clinically used antiarrhythmic agents have been shown to modulate K_2_P channels, suggesting that background K^+^ conductance may represent an underappreciated component of their electrophysiologic actions. Mexiletine (Class IB) and propafenone (Class IC), primarily recognized as sodium channel blockers, have demonstrated inhibitory effects on K_2_P channels such as TASK-1 and TREK-1 in experimental systems, which may contribute to atrial action potential modulation [76]. Carvedilol, a non-selective β-adrenergic blocker with additional antiarrhythmic properties, has also been shown to inhibit TASK-1 currents, suggesting a potential role for K_2_P modulation in its cardiac electrophysiologic effects [76]. On the other hand, current limitations in targeting K_2_P channels therapeutically include the poor pharmacological selectivity of available modulators, largely due to the structural similarity among K_2_P family members. This lack of specificity may lead to off-target effects, particularly given the widespread distribution of K_2_P channels throughout the cardiovascular and pulmonary systems. Additionally, these channels may be downregulated or suppressed by disease-associated factors such as hypoxia, extracellular pH changes, and GPCR signaling. Advances in precision pharmacology, including structure-guided drug design and gene-based therapeutic approaches targeting K_2_P or related pathways, may ultimately enable targeted restoration of membrane stability and vascular homeostasis in cardiopulmonary diseases. In parallel, the development of subtype-selective, tissue-targeted K_2_P modulators could enable precision therapies that restore membrane stability and vascular balance while minimizing off-target effects.

## Figures and Tables

**Table 1:** K_2_P Channels as Mediators of Cardiovascular and Pulmonary Disease Mechanisms and Pathophysiology.

K_2_P Channel/Subfamily	Associated Disease (s)	Pathophysiological Role/ Mechanism
TASK-1 (*KCNK3*)	Pulmonary arterial hypertension [37] (PAH); Atrial fibrillation (AF) [29]; Systemic hypertension [38]	Loss-of-function mutations cause membrane depolarization, vasoconstriction, and vascular remodeling. TASK-1 is upregulated in chronic AF and downregulated in AF with heart failure. TASK-1 SNPs are associated with systemic hypertension.
TASK-3 (*KCNK9)*	Atrial fibrillation [39]	May play a compensatory role in response to TASK-1 dysfunction.
TASK-4 (*KCNK17*)	Idiopathic ventricular fibrillation [40]	Gain-of-function mutations alter action potential repolarization, promoting reentrant arrhythmias.
TWIK-1 (*KCNK1*)	Chronic atrial fibrillation [41]	Contributes to inward rectifier K^+^ current in atrial and Purkinje fibers. Reduced TWIK-1 expression contributes to electrical remodeling.
TWIK-2 (*KCNK6*)	Pulmonary hypertension [11]; Systemic hypertension [42]	TWIK-2 knockout mice show PASMCs depolarization via the Rho kinase pathway, leading to vascular dysfunction and elevated blood pressure.
TREK-1 (*KCNK2*)	Ischemia–reperfusion injury [43]; Heart failure [44]; Pulmonary hypertension [45]; Hyperoxia- and influenza A-induced lung injury [46]	Downregulated in heart failure and AF. Activation is cardioprotective—limits Ca^2+^ overload, reduces PASMC remodeling, and protects against hyperoxia and viral lung injury.
TREK-2 (*KCNK10*)	Atrial fibrillation [47]; Heart failure [47]	Upregulation promotes electrical instability and reentry.

## Data Availability

No new data were created or analyzed in this study.

## Abbreviations

4TM/2P	4 Transmembrane/2 pore domain
K_2_P	Two-pore domain potassium channel
CVD	Cardiovascular Disease
ECC	Excitation–Contraction Coupling
GPCR	G-Protein-Coupled Receptors
PKA	Protein Kinase A
PKC	Protein Kinase C
PLC	Phospholipase C
[Ca^2+^]i	Intracellular Calcium Level
GHK	Goldman–Hodgkin–Katz equation
AF	Atrial Fibrillation
PASMCs	Pulmonary arterial smooth muscle cells
ECs	Endothelial cells
MCP-1	Monocyte chemoattractant protein-1
ARDS	Acute respiratory distress syndrome
PH	Pulmonary Hypertension
ET-1	Endothelin-1
ETA	Endothelin A receptor
DAG	Diacylglycerol
IP_3_	Inositol-1,4,5-trisphosphate
BP	Blood Pressure
